# Population Genetic Structure and Diversity of *Camellia oleifera*: Insights from Comparative Chloroplast Genomics and Development of Molecular Markers

**DOI:** 10.3390/biology15211955

**Published:** 2026-10-30

**Authors:** Linqin Xu, Xiaoyu Jiang, Junjie Liu, Lei Qi, Chenxi Yang, Haojun Li, Yao Huang, Lijun Zhou, Shiling Feng, Hongyu Yang, Chunbang Ding, Tao Chen

**Affiliations:** 1 College of Life Science, Sichuan Agricultural University, Ya’an Campus, Ya’an 625014, China; 18181002760@163.com (L.X.); jiangxyu1997@outlook.com (X.J.); 13350950617@163.com (J.L.); surrendereds@163.com (L.Q.); aa13322355268@163.com (C.Y.); lihaojun580@outlook.com (H.L.); 19354046368@163.com (Y.H.); zhoulijun@sicau.edu.cn (L.Z.); fsl@sicau.edu.cn (S.F.); yhy4868135@163.com (H.Y.); 2 Southwest Bio-Resources R&D Key Laboratory of Sichuan Province, Sichuan Agricultural University, Ya’an 625014, China

**Keywords:** *Camellia oleifera*, comparative genomics, genetic diversity, population genetic structure

## Abstract

This study aimed to develop chloroplast sequence markers through comparative plastome analysis and evaluate their application in haplotype identification and genetic diversity assessment in sampled *Camellia oleifera* populations. Three newly assembled chloroplast genomes of *Camellia* sect. *Oleifera* ranged from 156,918 to 157,003 bp and retained the typical quadripartite structure. Comparative analyses of these genomes and 13 reference plastomes identified 605 long repeats and 3315 SSRs. The three new plastomes shared 29 preferred sense codons, 28 ending in A/U. Sliding-window nucleotide diversity highlighted eight candidate variable regions, with a maximum π of 0.01397. Among 324 individuals from 37 sampling populations, the 1772-bp concatenated alignment contained 38 variable sites and 55 haplotypes (Hd = 0.8052; π = 0.002183). Thirty-six haplotypes were singletons, while H003 occurred in 133 individuals across 35 populations. Sequence PCA and exploratory ADMIXTURE described broad variation. K = 2 had the lowest cross-validation error among the values shown for K = 2–10, and its two components were interpreted descriptively. These results document chloroplast sequence diversity and population structure in the sampled *C. oleifera* material and provide molecular resources for germplasm characterization and conservation sampling.

## Simple Summary

Oil tea (*Camellia oleifera*) is cultivated for its edible seed oil. Understanding its genetic variation can support the conservation and use of genetic resources. Three chloroplast markers were developed and tested in 324 plants from 37 sampling populations. The 1772 bp alignment identified 55 chloroplast sequence types, including a common type shared across 35 populations and 36 types found in only one plant. These markers support genetic resource characterization and diversity assessment, although broader application requires further evaluation.

## 1. Introduction

*Camellia oleifera* C. Abel (*C. oleifera*), a mostly evergreen shrub or medium tree, belongs to the *Camellia* genus of the Theaceae family and grows primarily in the subtropical and warm-temperate regions [1,2]. *C. oleifera* is predominantly outcrossing, with late-acting self-incompatibility limiting self-fertilization [3]. As an important economic oilseed crop, *C. oleifera*, rich in oil and saponins, is widely cultivated in the southern subtropical regions of China and has a history of use spanning over two millennia [4,5]. From the perspective of economic value, the *C. oleifera* oil industry has become a crucial pillar for the coordinated promotion of rural revitalization, ecological protection, and economic development in southern China’s mountainous areas [6]. From an ecological perspective, *C. oleifera* demonstrates strong adaptability to nutrient-poor soils and high environmental stress resistance [7]. Ecological studies have examined carbon storage following reforestation, small-mammal responses to conversion to oil-tea plantations, and nutrient losses under intercropping [8,9,10]. However, with the large-scale expansion of the *C. oleifera* oil industry and the extensive adoption of artificially selected cultivars, natural *C. oleifera* resources are facing two critical challenges. First, habitat fragmentation, over-excavation, and anthropogenic disturbances have precipitated a pronounced decline in the population size of wild *C. oleifera*, thereby increasing the risk of genetic resource erosion [11,12]. Second, the limited exploration of the genetic background of wild populations in current breeding programs has led to a restricted genetic base in cultivated varieties [13], thereby impeding improvements in stress resistance (e.g., salt–alkali and drought tolerance) and overall quality. Consequently, a systematic analysis of the genetic diversity, genetic structure, and evolutionary dynamics of natural *C. oleifera* populations is imperative: not only for the conservation of genetic resources in this ecologically and economically significant woody oil species but also as a fundamental scientific endeavor to facilitate sustainable breeding within the *C. oleifera* industry.

Population genetic structure and diversity are fundamental indicators of a species’ environmental adaptability, evolutionary potential, and value for resource utilization [14,15,16]. Chloroplast and mitochondrial genomes provide complementary resources for investigating organellar genome organization and evolutionary relationships in *Camellia*. For example, Zhang et al. [17] assembled the chloroplast and mitochondrial genomes of *C. sinensis* var. *assamica*, while Gu et al. [18] characterized the mitochondrial genome of *C. oleifera*, examining its gene composition, conserved gene clusters, and structural variation. These studies advance the understanding of organellar genome evolution in the genus. The present study focuses on comparative chloroplast genomics and the development of chloroplast sequence markers for assessing intraspecific genetic diversity in *C. oleifera*.

The chloroplast genome, as a maternally inherited organellar genome, is characterized by structural conservation, a moderate evolutionary rate, and abundant variation in single-copy regions [19], making it an optimal molecular marker for plant population genetic studies. Despite this overall conservation, expansion and contraction at the boundaries between inverted repeats and single-copy regions can contribute to variation in chloroplast genome size [20,21]. In addition, repeats in chloroplast genomes are associated with genome rearrangement and sequence variation [22], and comparisons of repeats and other variable features can inform the selection of candidate molecular markers [23,24]. Numerous studies have shown that coding regions of the angiosperm chloroplast genome are more conserved than non-coding regions, which therefore exhibit higher nucleotide polymorphism [25,26,27]. Accordingly, regions such as *trnL-trnF* and *psbA-trnH* are particularly useful for investigating chloroplast sequence variation among populations and developing molecular markers [28,29]. Within the genus *Camellia*, chloroplast genome studies have yielded important insights into the origin and evolution of tea plants (*C. sinensis*) and the taxonomic identification of oil tea-related species (e.g., *C. vietnamensis* and *C. meiocarpa*) [25,30,31]. Building on previous population genetic studies [32,33], the present study evaluates chloroplast markers selected through comparative plastome analysis for characterizing sequence variation within *C. oleifera*.

Despite the necessity of molecular markers for precise identification and utilization of *C. oleifera* genetic resources, existing tools such as ISSRs and RAPDs are constrained by questionable homology assessments, poor repeatability, and insufficient coverage [34]. Markers derived from highly variable chloroplast genome regions can be used to characterize chloroplast sequence variation, with potential applications in population tracing and germplasm verification [35,36]. The application of these markers to intraspecific studies requires evaluation of the sequence variation they detect in the target material. The selected chloroplast markers were therefore assessed for haplotype identification and genetic diversity assessment in the sampled *C. oleifera* individuals.

This study aimed to compare the genome structure and sequence variation of three newly assembled chloroplast genomes and 13 reference plastomes, develop and evaluate three chloroplast sequence markers for detecting intraspecific variation in *C. oleifera*, and describe haplotype relationships and major patterns of chloroplast sequence variation among the sampled individuals using haplotype networks, sequence PCA, and exploratory clustering.

## 2. Materials and Methods

### 2.1. Plant Material Collection

Total genomic DNA was extracted from three plant samples, and their complete chloroplast genomes were assembled and annotated. YC127 (*C. oleifera*) and XYYC (*C. lanceoleosa*) were collected from the International *Camellia* Species Garden in Jinhua, Zhejiang Province, China (29.119600° N, 119.597746° E), with voucher numbers 2024062 and 2024042, respectively. HNL2-2 (*C. oleifera*) was collected from Tianquan County, Ya’an, Sichuan Province, China (30.066706° N, 102.758386° E), with voucher number 2024057. Plant material was collected with permission from the relevant site managers. All voucher specimens (Figure S1) were deposited in the Herbarium of the College of Life Science, Sichuan Agricultural University.

**Figure 1:**
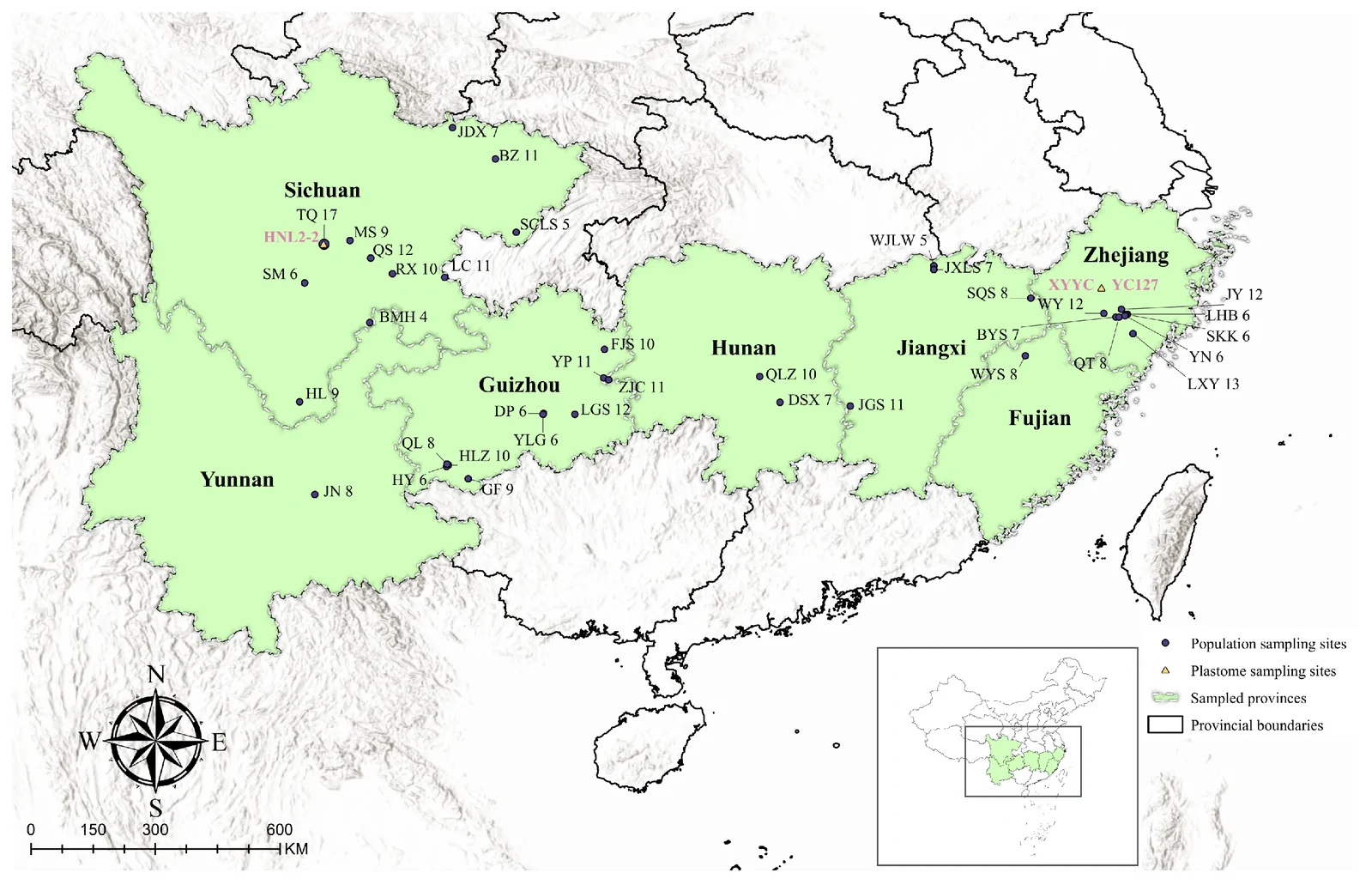
Sampling locations of 37 *C. oleifera* populations (324 individuals). Numbers indicate analyzed sample sizes. Triangles mark plastome samples: YC127 and XYYC from Jinhua, Zhejiang, and HNL2-2 from Tianquan, Sichuan.

A total of 337 *C. oleifera* individuals were collected. The population analyses retained 324 individuals with sequences available for all three chloroplast markers, representing 37 sampling populations across seven provinces; 13 individuals were excluded from this complete-marker dataset. The retained populations comprised 11 in Sichuan (BMH, BZ, HL, JDX, LC, SCLS, MS, QS, RX, SM, and TQ), 10 in Guizhou (DP, FJS, GF, HLZ, HY, LGS, QL, YLG, YP, and ZJC), eight in Zhejiang (BYS, JY, LHB, LXY, QT, SKK, WY, and YN), four in Jiangxi (JGS, JXLS, SQS, and WJLW), two in Hunan (DSX and QLZ), one in Yunnan (JN), and one in Fujian (WYS). Sampled plants were separated by more than 10 m. Ten healthy leaves per individual were washed with 75% ethanol (Chron, Chengdu, China) and stored in silica gel (Chron, Chengdu, China). Sampling locations and retained sample sizes are shown in Figure 1 and Supplementary Table S1.

All leaf samples analyzed in this study were newly collected. The recorded coordinates of 27 sampling localities (Nos. 1–27 in Supplementary Table S1) were identical to or close to those reported by Chen et al. (2025) [33]. The remaining 10 sampling localities (Nos. 28–37) provided additional sampling coverage in Jiangxi, Fujian, and Sichuan.

### 2.2. Chloroplast Genome Extraction, Sequencing, Assembly, and Annotation

Total genomic DNA was extracted using a modified protocol based on cetyltrimethylammonium bromide (CTAB) [37]. DNA quality was evaluated through electrophoresis on a 1% agarose gel, and its concentration was determined using a NanoDrop 2000C UV-Vis Spectrophotometer (Thermo Fisher Scientific, Wilmington, DE, USA). Sequencing libraries were prepared using VAHTS^®^ Universal DNA Library Prep Kits (VAZYME, Nanjing, China) for Illumina V3. PE150 sequencing was performed on an Illumina HiSeq 4000 platform by BGI Genomics (Shenzhen, China).

Raw reads were filtered using fastp v0.20.0 [38]. Chloroplast genomes were assembled de novo using GetOrganelle v1.7.7.0 [39] in embryophyte plastid mode (-F embplant_pt), with a maximum of 10 rounds of read extension (-R 10) and SPAdes k-mer sizes of 21, 45, 65, 85, and 105. Assemblies were run using one thread (-t 1). Quality-filtered paired-end reads were mapped to the corresponding plastome assemblies using BWA-MEM v0.7.19-r1273 [40]. Mean coverage depth across the entire plastome was calculated using SAMtools v1.24 [41]. Genome annotation was performed using CPGAVAS2 [42], with the published *Camellia oleifera* chloroplast genome MN078090.1 as a reference, followed by manual inspection and correction. Gene names, coding-region boundaries, exon structures, and pseudogenes were determined based on sequence evidence and comparative annotation. Gene counts included annotated CDS loci, and tRNA and rRNA features. Unique genes were counted by grouping copies with the same gene name within each feature class.

### 2.3. Genome Structure and Repeat Sequence Analysis

Thirteen previously reported *Camellia* chloroplast genomes were obtained from NCBI and analyzed together with the three new plastomes (Table 1). GC content was calculated using Biopython v1.84 [43]. Repeat sequences were searched using Vmatch v2.3.1 through the repfind.pl compatibility script for REPuter [44], with a minimum size of 30 bp and a maximum Hamming distance of 3 between the two repeats. Simple sequence repeats were analyzed using CPStools v3.6.1 [45], with minimum repeat numbers of 8, 4, 4, 3, 3, and 3 for mono-, di-, tri-, tetra-, penta-, and hexanucleotide motifs, respectively. IR/SC boundaries were compared using IRscope v0.1 [46]. Plastome regions were designated LSC–IRb–SSC–IRa.

### 2.4. Codon Usage Bias Analyses

Relative synonymous codon usage (RSCU) was calculated for the three newly assembled plastomes (YC127, XYYC, and HNL2-2) using CPStools v3.6.1 [45]. CDSs were extracted in transcriptional order, accounting for joined exons and codon_start. Sequences were selected using the following criteria: (1) an ATG start codon and no internal stop codons; (2) a minimum length of 300 bp; and (3) a length divisible by three [47]. One qualifying copy was retained per gene name in each plastome. Preferred sense codons were defined as those with RSCU > 1. RSCU plots were generated using the CPStools plotting module. Codon counts and RSCU values are provided in Supplementary Table S6.

### 2.5. Nucleotide Variation Analysis and Development of Hypervariable Molecular Markers

The three new plastomes and 13 reference plastomes were aligned using MAFFT v7.526 (--auto, four threads) [48]. Sliding-window nucleotide diversity (π) was calculated using CPStools v3.6.1 [45], with a window length of 600 alignment columns and a step of 100 columns [49]. An additional full window was anchored at the alignment end. Columns containing a gap or non-ACGT character in any sequence were excluded within each window. Windows with fewer than 50 retained sites or less than 50% callable columns were excluded. XYYC was used for coordinate mapping and annotation. Candidate windows were identified using the 95th percentile of valid-window π (0.00287270) as the cutoff and grouped by their positions and annotations.

Marker1 overlaps *trnG-UCC*, marker2 lies within *ndhF*, and marker3 spans the *trnL-UAA*–*trnF-GAA* region and adjacent sequence, including *trnF-GAA* and part of the downstream interval. Primers were designed using Primer Premier 6.25. PCR used a 20 µL reaction containing 10 µL of 2 × PCR Mix (Solarbio, Beijing, China), 1 µL of each primer (10 µM), 1 µL of genomic DNA, and 7 µL of water. Cycling comprised 94 °C for 3 min; 35 cycles of 94 °C for 30 s, 50–55 °C for 30 s, and 72 °C for 60 s; and 72 °C for 5 min. Products were purified and sequenced by Sangon Biotech (Shanghai, China).

### 2.6. Genetic Diversity Calculation and Genetic Structure Analysis

Population analyses included 324 individuals from 37 sampling populations. The three chloroplast fragments were aligned in a common orientation, trimmed, and concatenated. The source alignment (combined_data324_trimmed2.fas; Supplementary File) contained 1796 columns. Complete deletion of 24 columns containing a gap in any individual yielded a 1772 bp alignment comprising 589, 544, and 639 bp from markers 1–3, respectively. The concatenated fragments were analyzed as one haploid chloroplast sequence.

Distinct fragment sequences were treated as marker alleles. The number of alleles (Na), effective number of alleles (Ne), Shannon information index (I), diversity (h), and polymorphic information content (PIC) were calculated from pooled fragment-haplotype frequencies in Python v3.12.14, following the definitions used in GenAlEx 6.5 [50] and Cervus v3.0 [51]: Ne = 1/Σp_i_^2^, I = −Σp_i_ ln(p_i_), h = 1 − Σp_i_^2^, and PIC = 1 − Σp_i_^2^ − 2Σ_i_ < _j_p_i_^2^p_j_^2^. For the concatenated sequences, the number of haplotypes (NH), segregating sites (S), haplotype diversity (Hd), and nucleotide diversity (π) were calculated from the complete alignment. Hd included the finite-sample correction *n*/(*n* − 1), and π was the mean pairwise nucleotide difference divided by 1772. A haplotype network was constructed in PopART v1.7 [52] using the Minimum Spanning Network algorithm with epsilon set to 0. Haplotype identifiers, states at 38 variable sites in the 55 chloroplast haplotypes, frequencies, and population composition are provided in Supplementary Table S5.

Sequence PCA used 84 nucleotide-state indicator variables representing all 38 variable sites. Variables were centered without variance scaling, and axis percentages were calculated from the full variance spectrum. The NJ tree was constructed using the complete 1772 bp alignment in MEGA-CC v11.0.13 [53], with p-distances, complete deletion, and 1000 bootstrap replicates. The tree was displayed as a cladogram with aligned tips.

Population structure was explored using ADMIXTURE v1.4.0 [54] in haploid mode, based on 33 biallelic variable sites extracted from the concatenated cpDNA alignment. Five-fold cross-validation was used to evaluate K values from 2 to 10. Additional haploid-mode runs used ten seeds per K to assess sensitivity to random initialization. The K = 2 model was used to describe the main coefficient pattern; individuals were assigned to the component with the higher Q value. Population labels below the coefficient plot were assigned according to the majority of individuals within each population. These exploratory components were not treated as independently established biological populations.

## 3. Results

### 3.1. Chloroplast Genome Characteristics

**Figure 2:**
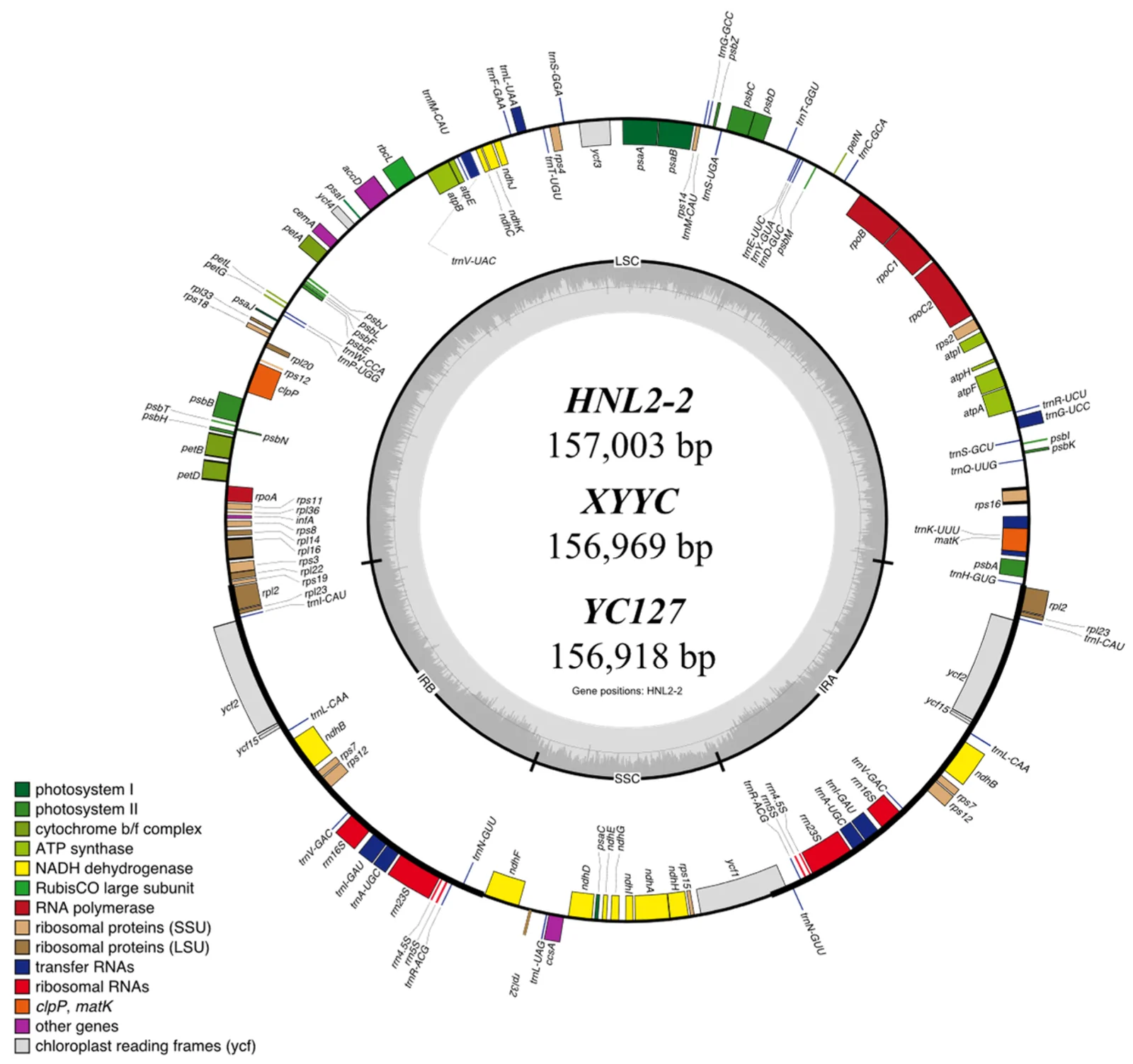
Representative chloroplast gene map of HNL2-2. YC127 and HNL2-2 are *C. oleifera* samples, and XYYC is a *C. lanceoleosa* sample. Gene functions are distinguished by color. The center lists the sizes of HNL2-2, XYYC, and YC127; gene positions correspond to HNL2-2. The grey inner circle shows its GC distribution.

The numbers of clean reads for HNL2-2, XYYC and YC127 were 31,934,622, 21,705,364 and 27,201,444, respectively, counting both mates separately. The corresponding mean plastome coverage depths were 69.33×, 102.25× and 69.06×. The proportions of plastome positions covered by at least one read were 100% for all three samples. The three new plastomes were 156,918 bp (YC127), 156,969 bp (XYYC), and 157,003 bp (HNL2-2), with the conserved quadripartite organization typical of *Camellia* [55]. Across all 16 plastomes, lengths ranged from 156,512 to 157,089 bp, a span of 577 bp (Table 1). LSC, SSC, and single-copy IR lengths ranged from 86,224 to 86,659 bp, 18,132 to 18,408 bp, and 25,943 to 26,165 bp, respectively. Total GC content ranged from 37.28% to 37.33%. In the three new plastomes, GC content was higher in the IRs (42.98–43.02%) than in the LSC (35.29–35.31%) and SSC (30.54–30.55%), consistent with the enrichment of rRNA and tRNA loci in the IRs [56,57]. Figure 2 uses HNL2-2 as a representative gene-position map.

Each of the three new plastomes contained 132 annotated gene loci, comprising 87 CDS loci, 37 tRNAs, and eight rRNAs, representing 80, 30, and four unique genes, respectively. Seven protein-coding genes (*ndhB*, *rpl2*, *rpl23*, *rps7*, *rps12*, *ycf2*, and *ycf15*), seven tRNA genes (*trnA-UGC*, *trnI-CAU*, *trnI-GAU*, *trnL-CAA*, *trnN-GUU*, *trnR-ACG*, and *trnV-GAC*), and four rRNA genes had duplicated copies. Nine protein-coding genes contained one cis-spliced intron (*atpF*, *ndhA*, *ndhB*, *petB*, *petD*, *rpl2*, *rpl16*, *rpoC1*, and *rps16*), whereas *clpP* and *ycf3* each contained two. The trans-spliced *rps12* annotations comprised three exons, with one trans-spliced and one cis-spliced intron. Six tRNA gene types also contained an intron (Supplementary Tables S2 and S3).

### 3.2. Repeat Sequences and IR/SC Boundary Comparisons

**Figure 3:**
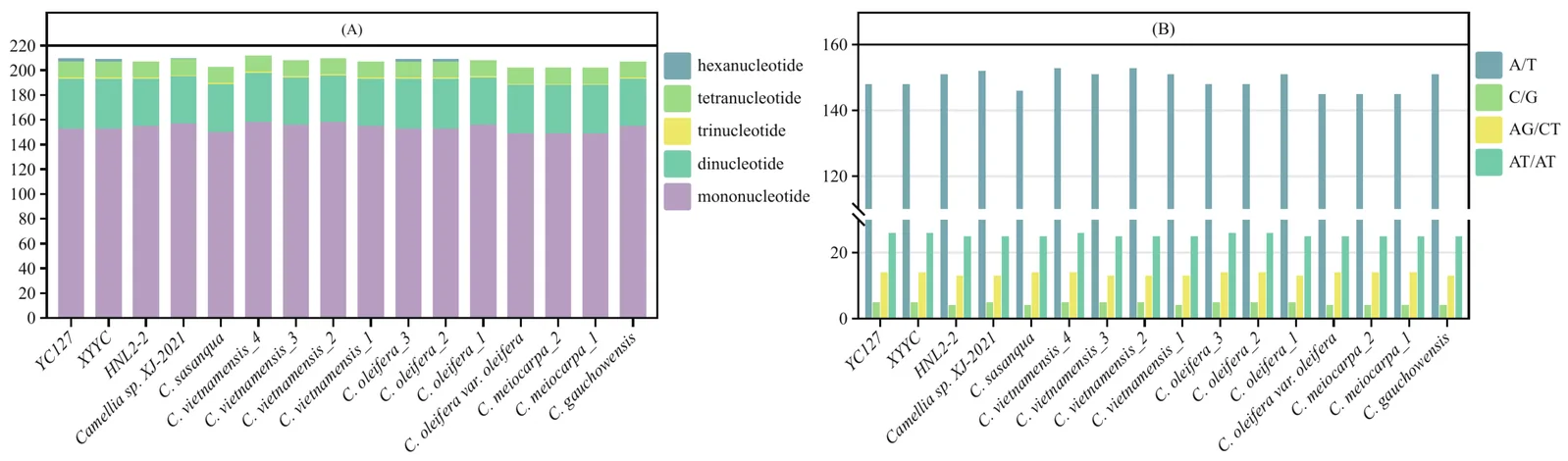
Analyses of the SSRs in 16 Sect. *Oleifera* chloroplast genomes: (**A**) Numbers of different SSR types. (**B**) Numbers of main repeat units of SSRs.

A total of 3315 SSRs were identified across the 16 plastomes (Figure 3; Supplementary Table S4). Mononucleotide repeats were the most abundant class (2459; 74.18%), followed by dinucleotide (622), tetranucleotide (208), trinucleotide (16), and hexanucleotide repeats (10); no pentanucleotide repeats were recorded. A/T-rich motifs predominated.

**Figure 4:**
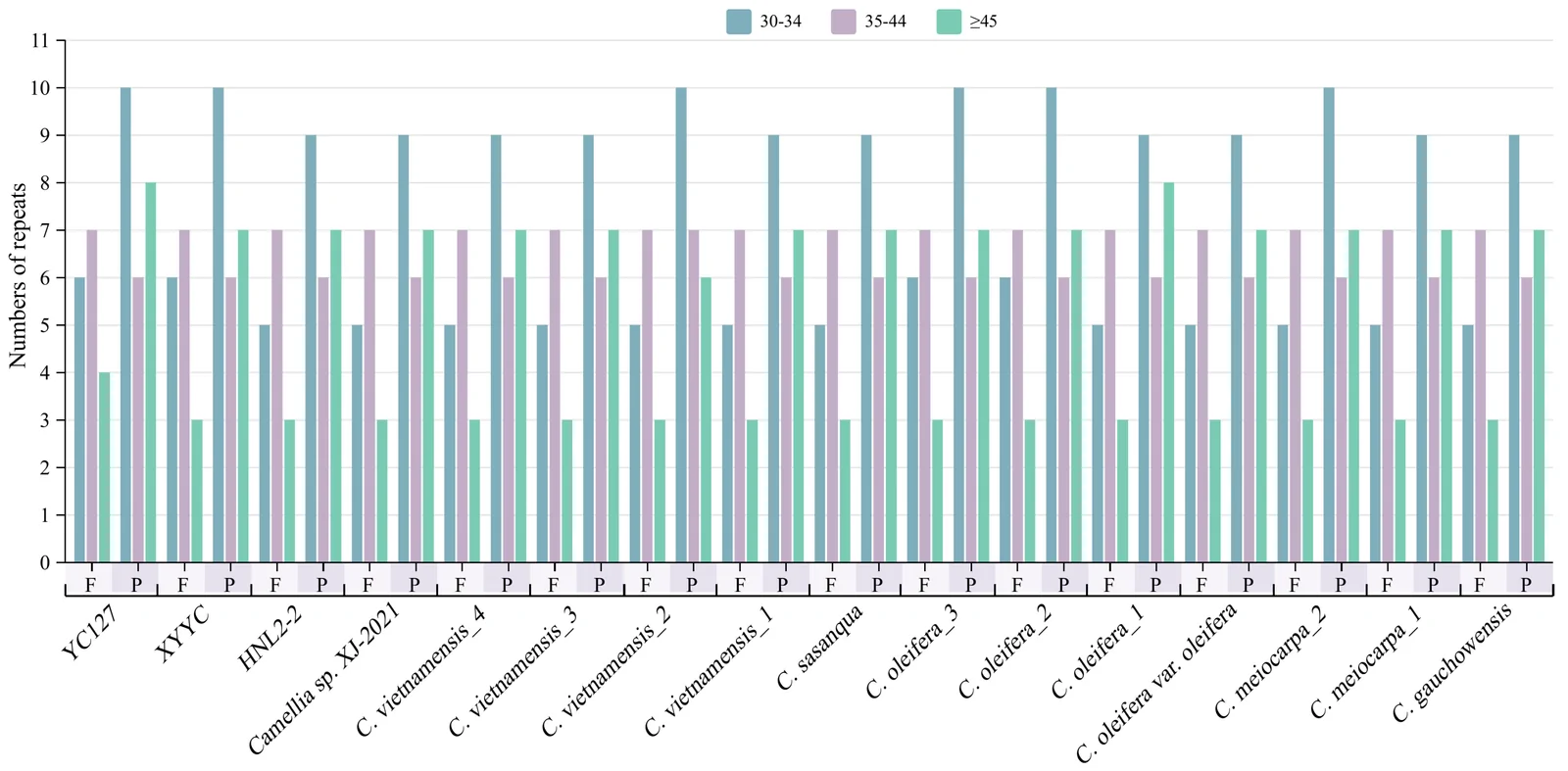
Long repeats in 16 *Camellia* plastomes, showing 605 repeats. F and P indicate forward and palindromic repeats. Colors identify non-overlapping repeat-length classes of 30–34, 35–44 and ≥45 bp.

A total of 605 long repeats were identified across the 16 plastomes, comprising 245 forward and 360 palindromic repeats (Figure 4). The non-overlapping length classes contained 234 repeats of 30–34 bp, 209 of 35–44 bp, and 162 of at least 45 bp. The 30–34-bp class was the most frequent (38.68%).

**Figure 5:**
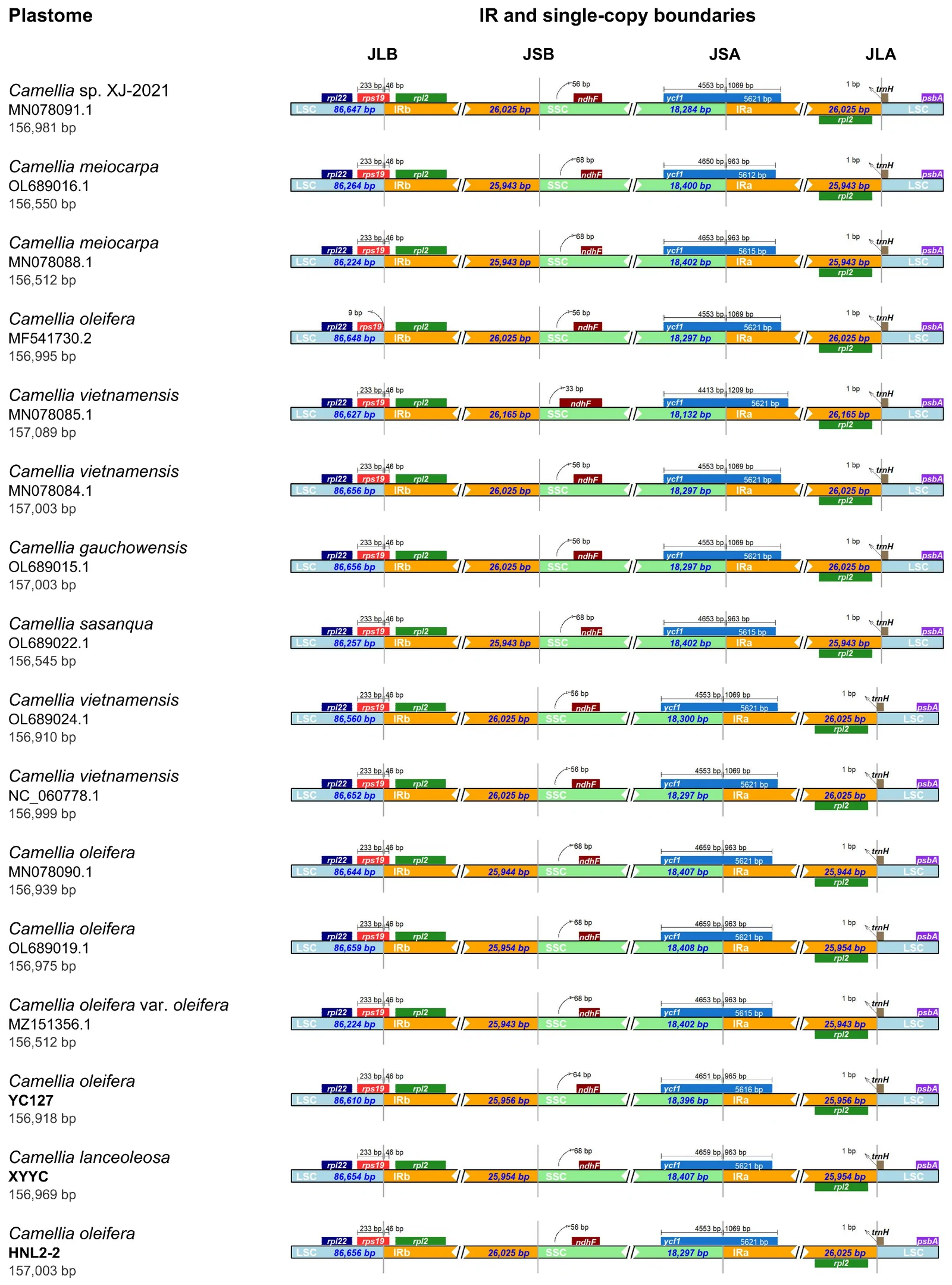
IR and single-copy boundary comparison across 16 *Camellia* plastomes, arranged as LSC-IRb-SSC-IRa. The positions and lengths of genes adjacent to each boundary are indicated.

The boundary comparison showed the LSC–IRb–SSC–IRa organization across the 16 plastomes (Figure 5). The *ycf1* gene crossed the SSC–IRa junction in all plastomes, extending 963–1209 bp into IRa. The *rps19* gene crossed the LSC–IRb junction in 15 plastomes but remained entirely within the LSC in *C. oleifera* MF541730.2.

### 3.3. Codon Usage Pattern

**Figure 6:**
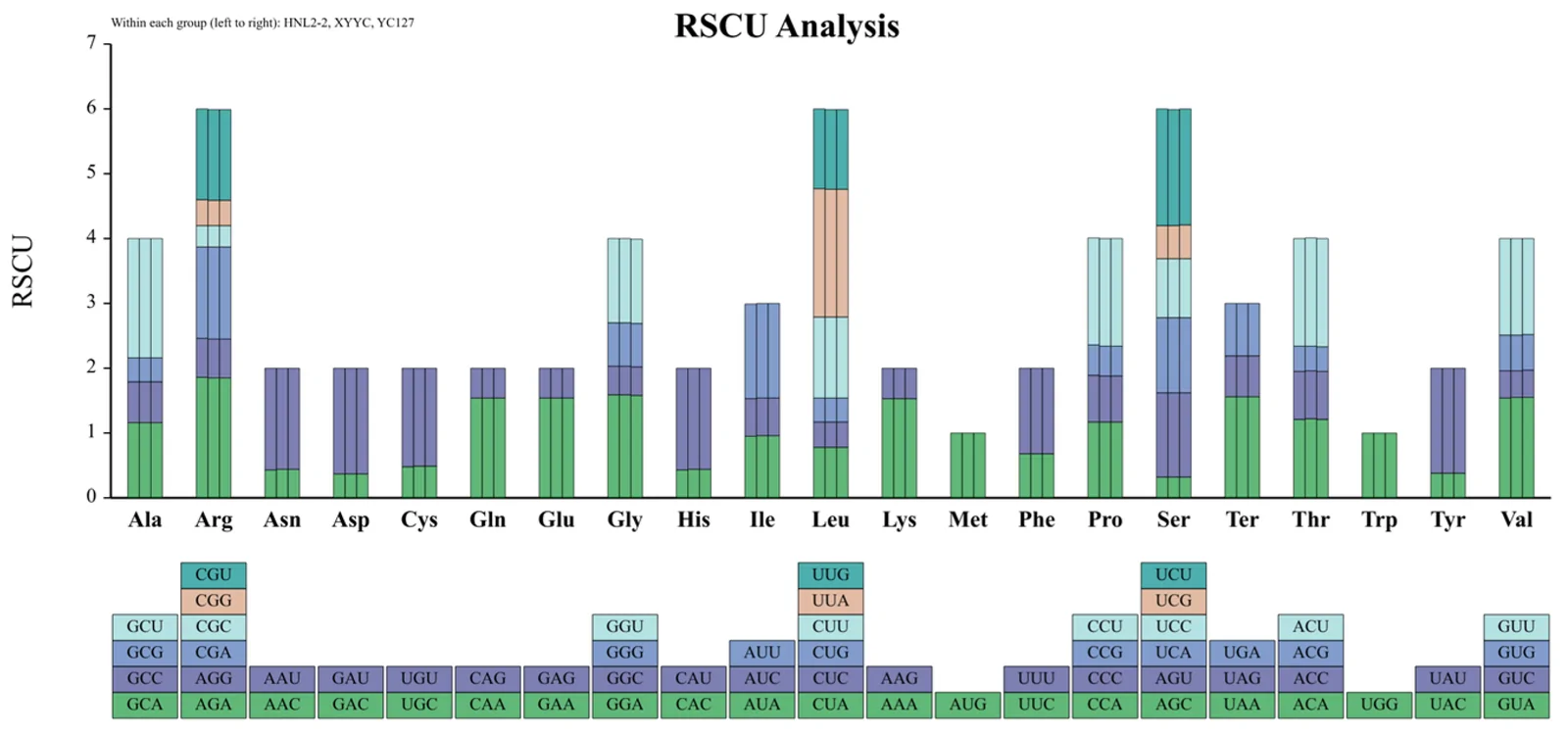
Relative synonymous codon usage (RSCU) in the three new plastomes. Within each amino acid group, bars represent HNL2-2, XYYC, and YC127 from left to right. Colored segments identify synonymous codons, shown in RNA notation. Ter denotes stop codons, which are excluded from the preferred-sense-codon count. Stacked height is the sum of RSCU values within each synonymous family.

Codon usage was compared among the three new plastomes using RSCU. Across the 61 sense codons, all three samples shared 29 codons with unrounded RSCU > 1. Of these, 28 ended in A or U; UUG was the exception. Terminal stop codons were displayed separately and excluded from the preferred-sense-codon count. The overall patterns were similar among samples (Figure 6).

### 3.4. Nucleotide Diversity and Candidate Variable Regions

**Figure 7:**
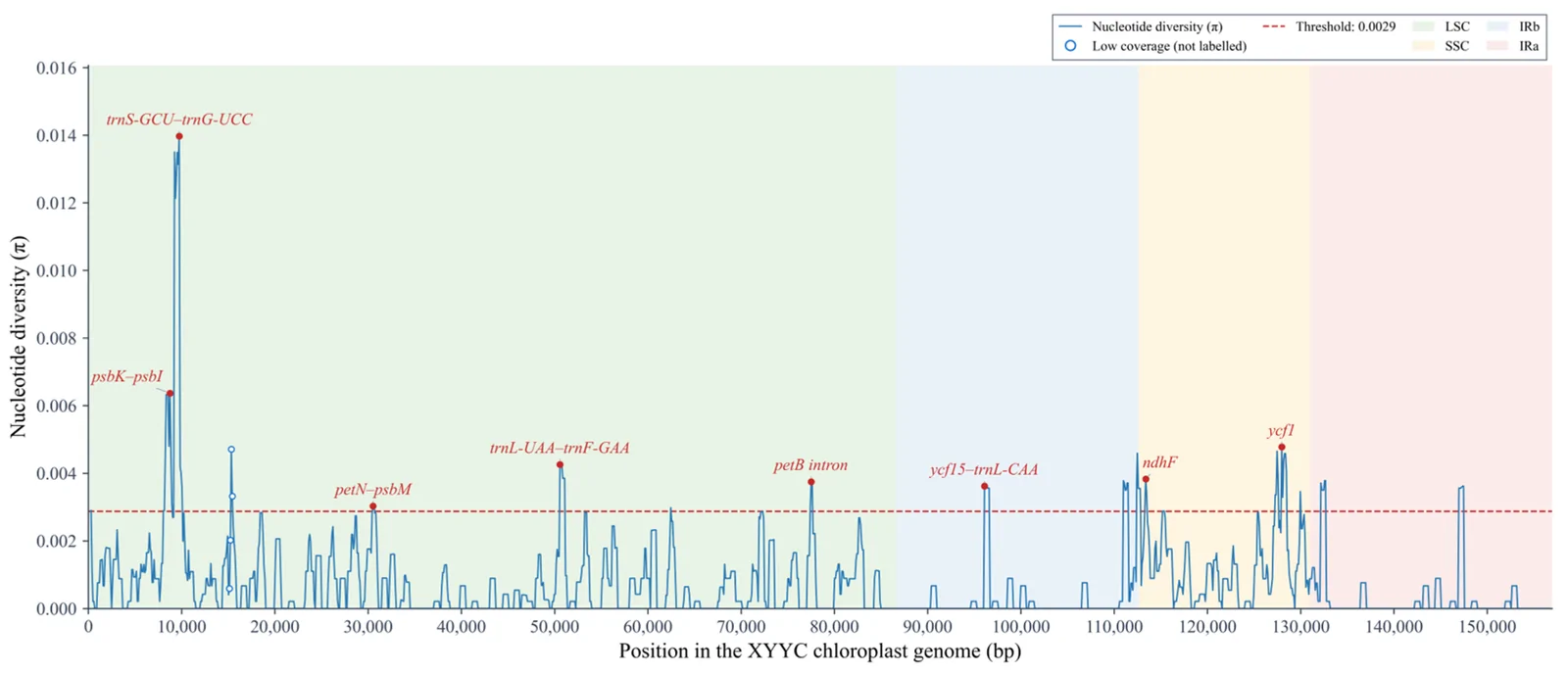
Sliding-window nucleotide diversity (π) across all 16 plastomes, using 600 alignment columns per window and a 100-column step. The *x*-axis shows mapped XYYC coordinates; background colors identify LSC, IRb, SSC, and IRa. The dashed line marks the 95th-percentile cutoff (π = 0.00287270). Eight selected variable regions are highlighted; empty points identify low-coverage windows excluded from screening.

The 16-plastome alignment contained 157,493 columns, including 156,009 sites callable across all samples. Of 1570 windows, 1566 met the coverage criteria. Valid-window π ranged from 0 to 0.01397 (Figure 7). The highest window mapped to XYYC positions 9444–10,043 and overlapped *trnS-GCU*–*trnG-UCC* and the adjacent *trnG-UCC* gene. The 95th-percentile cutoff identified 79 windows in 17 contiguous groups. Figure 7 highlights eight selected variable regions: *trnS-GCU*–*trnG-UCC*, *psbK*–*psbI*, *petN*–*psbM*, *trnL-UAA*–*trnF-GAA*, the *petB* intron, *ycf15*–*trnL-CAA*, *ndhF*, and the SSC *ycf1* copy. Regional variation included the marker targets and additional candidate intervals. Site-based regional π was lower in IRb (0.00028471) and IRa (0.00028953) than in the LSC (0.00086727) and SSC (0.00132737).

### 3.5. Genetic Diversity

The three markers detected 9, 31, and 8 distinct fragment haplotypes for marker 1, marker 2, and marker 3, respectively (mean Na = 16.0; Table 2). PIC ranged from 0.3788 for marker 3 to 0.6764 for marker 2, with a mean of 0.4789. Mean Ne, I, and h were 2.2863, 1.1667, and 0.5267, respectively. Marker 2 had the greatest sequence-type richness and frequency-based diversity within the retained sample set, whereas markers 1 and 3 had lower diversity and similar PIC values. The markers therefore differed in their level of polymorphism, with marker 2 providing the most variation for chloroplast haplotype characterization in these samples.

The 1772 bp concatenated alignment of 324 individuals contained 38 segregating sites and 55 haplotypes. Overall, Hd was 0.8052, and π was 0.002183, equivalent to an average of 3.8674 pairwise nucleotide differences. Among the 37 populations, sample sizes ranged from four to 17, Hd from 0.4286 to 1.0000, and π from 0.000376 to 0.003601 (Table 3). HL, MS, QLZ, TQ, and WY each contained seven haplotypes, the highest observed count. BMH and SCLS had Hd = 1.0000, whereas DSX had the highest π (0.003601). Population rankings therefore differed between haplotype richness and nucleotide diversity.

### 3.6. cpDNA Haplotype

**Figure 8:**
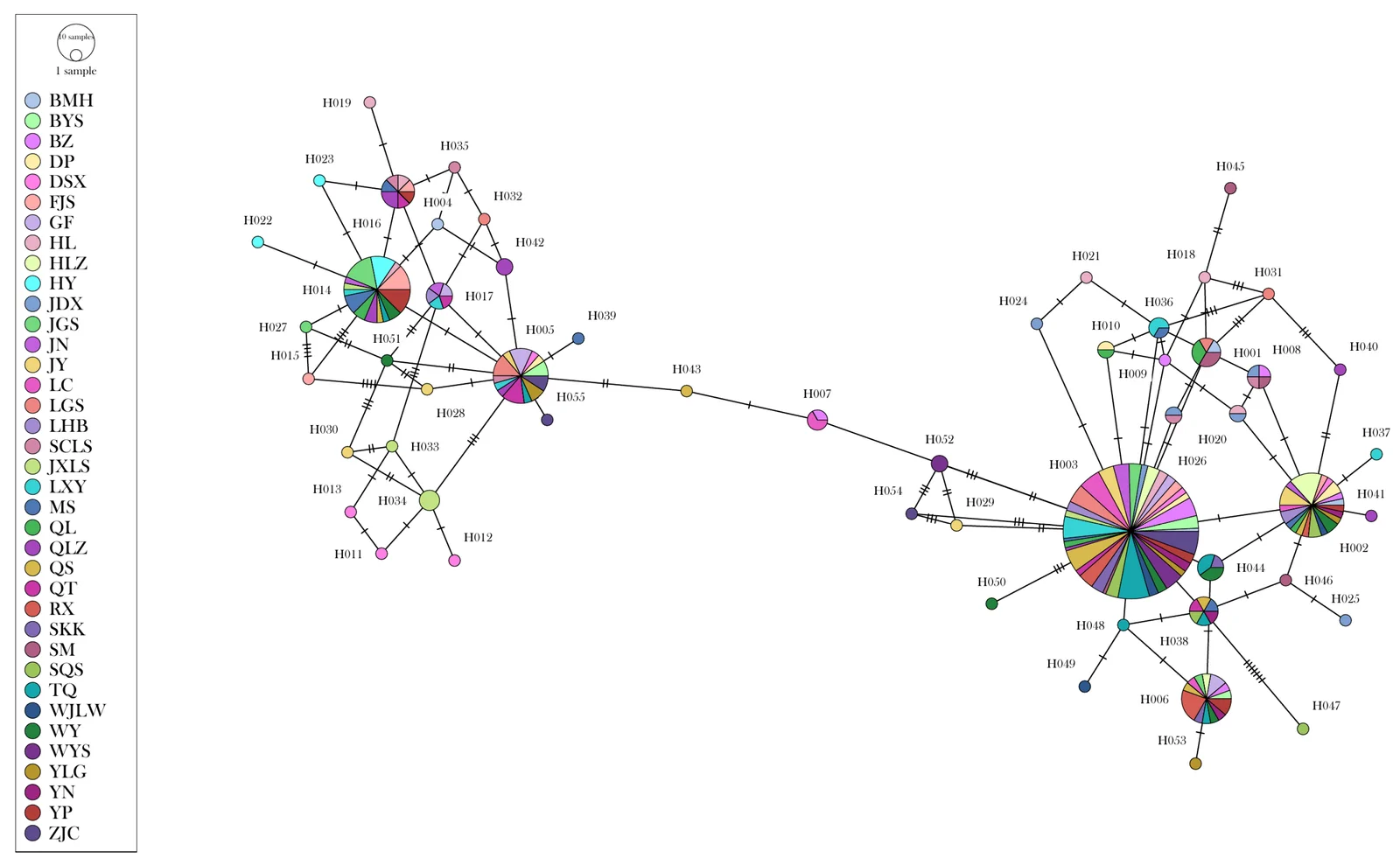
Network of 55 chloroplast haplotypes identified in the 1772 bp concatenated cpDNA alignment of 324 individuals from 37 sampled populations. Circle size is proportional to haplotype frequency, and colored sectors indicate the composition of sampled populations. Haplotype identifiers H001–H055 correspond to Supplementary Table S5; 36 haplotypes are singletons. H003 and H014 occur in 133 and 32 individuals, respectively. Each hatch mark on a connecting line represents one mutational step (one nucleotide difference) between haplotypes.

As shown in Figure 8, the network contained 55 haplotypes from 324 individuals in 37 sampling populations. Thirty-six haplotypes occurred in a single individual, whereas the remaining 19 occurred in two or more individuals. H003 was the most frequent haplotype, occurring in 133 individuals across 35 populations. H014 was the second most frequent haplotype, occurring in 32 individuals across 14 populations. Common haplotypes were shared among sampling populations, while many low-frequency haplotypes represented finer sequence differences. Haplotype frequencies and population composition are provided in Supplementary Table S5.

### 3.7. Genetic Structure Based on Chloroplast DNA

**Figure 9:**
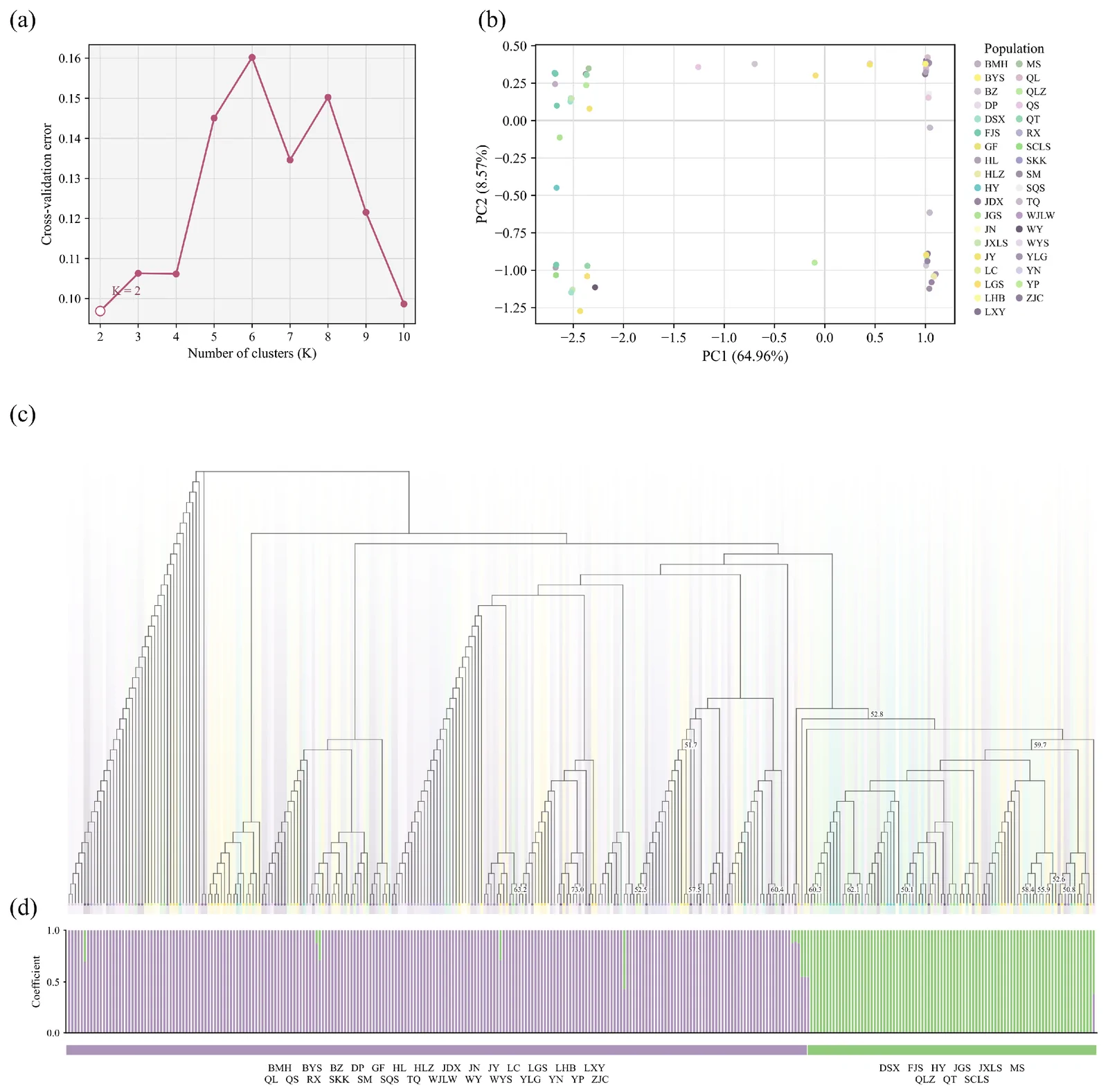
Chloroplast sequence variation and exploratory population structure among 324 individuals from 37 sampling populations: (**a**) Five-fold ADMIXTURE CV error for K = 2–10, with K = 2 highlighted as the minimum within this displayed range. (**b**) Sequence PCA from all 38 variable sites; PC1 and PC2 explain 64.96% and 8.57% of the variance, respectively, and colors identify sampling populations. (**c**) NJ cladogram from the complete 1772 bp alignment, using p-distances and 1000 bootstrap replicates. Positive-length internal branches with support ≥50% are labeled. Colored tails identify sampling populations. (**d**) ADMIXTURE K = 2 coefficients from 33 biallelic sites, with individual order matching the NJ tree. The lower two-color strip shows the two model components; each population name is listed once according to its majority maximum-Q assignment.

Sequence PCA explained 64.96% and 8.57% of total variance on PC1 and PC2, respectively (73.53% cumulatively; Figure 9b). Individuals mainly occupied two regions along PC1, with a small number of intermediate positions. Individuals from different sampling populations overlapped in both regions of the ordination space.

Among the K values shown in the five-fold ADMIXTURE cross-validation comparison (K = 2–10), K = 2 had the lowest error (Figure 9a). The K = 2 coefficients are shown in the same individual order as the NJ tips (Figure 9c,d) and describe two predominant coefficient patterns. Individuals from the same sampling population occurred in both components. The NJ tree provided limited support for many internal relationships; the displayed bootstrap values ranged from 50.1% to 73.0%. The two model components were therefore used as descriptive summaries of chloroplast sequence variation.

## 4. Discussion

### 4.1. Comparison of Chloroplast Genomes in the Sect. Oleifera

The 16 plastomes showed limited variation in genome size and retained the characteristic quadripartite organization of *Camellia*. Genome sizes differed by 577 bp across all 16 accessions and by 85 bp among the three newly assembled plastomes, which each contained 132 annotated gene loci. This agrees with the conserved genome structure and gene order reported by Huang et al. [55]. Their comparison also documented variation at IR–single-copy junctions, showing that small boundary differences can coexist with an otherwise conserved organization. Similar coexistence of structural conservation and IR-boundary variation was reported for the *Sinodielsia* clade [58]. The present boundary comparisons likewise place the size differences within a broadly conserved plastome framework.

The 605 long repeats and 3315 SSRs identified across the 16 plastomes provide additional resources for comparative analysis and marker development. Mononucleotide repeats accounted for 74.18% of SSRs, with A/T-rich motifs predominant. Such repeats can vary in copy number through replication slippage, providing a potential source of length polymorphism, as discussed in previous *Camellia* plastome research [55]. Their abundance identifies candidate loci for further polymorphism screening. The shared preference for 29 sense codons, including 28 ending in A/U, indicates similar codon-use patterns among the three new plastomes. A preference for A/U-ending codons has also been reported in other plant studies [59,60].

Sequence variation was unevenly distributed, with lower regional π in the IRs than in the LSC and SSC. Huang et al. [55] similarly found that *Camellia* IRs were more conserved than single-copy regions, while noncoding regions were generally more variable than coding regions. The eight selected variable regions highlighted in Figure 7 illustrate how localized sequence differences can provide marker targets despite overall structural conservation. The three developed markers cover intronic, coding, and intergenic sequences. Notably, the *ndhF* marker had the highest fragment-haplotype diversity, showing that a coding region can still contain useful intraspecific variation. Marker performance consequently depends on variation within the selected fragment, in addition to its genomic annotation.

### 4.2. Genetic Diversity Detected by Chloroplast Markers

The sequence variation detected by the three cpDNA markers developed from highly variable chloroplast regions supports their use for chloroplast haplotype identification and genetic diversity assessment in the sampled *C. oleifera* material. PIC was used as an auxiliary frequency-based description of marker polymorphism and was not interpreted as within-individual heterozygosity or evidence of population discrimination. Liang et al. [31] also used comparative plastome data to develop markers for *C. oleifera* and *C. meiocarpa*, detecting target-sequence variation with 17 of 56 primer pairs. That study evaluated differences between closely related species, whereas the present study demonstrates the value of this approach for detecting sequence variation among individuals within *C. oleifera*.

The 324 individuals from 37 populations contained 55 haplotypes, with Hd = 0.8052 and π = 0.002183. The coexistence of a widespread common haplotype and many rare haplotypes indicates that haplotype richness does not imply large sequence differences among all individuals. Chen et al. [33] identified 47 chloroplast haplotypes among 389 *C. oleifera* individuals using *rbcL* and *psbA–trnH*, with Hd = 0.771 and π = 0.00338. The present dataset contained more haplotypes and had a somewhat higher Hd but a lower π than that study, indicating fewer average per-site nucleotide differences despite the larger number of sequence types. Qi et al. [32] reported Hd = 0.978 and π = 0.00413 for *C. oleifera* based on five chloroplast fragments, both numerically higher than the corresponding estimates in the present study. Studies of related species provide further context. For example, Li et al. [61] detected eight haplotypes in 120 *C. huana* individuals across 5200 bp, with Hd = 0.759 and π = 0.00042, both lower than in the present study. However, differences in the chloroplast fragments analyzed, sequence lengths, and sampling designs limit direct comparisons of overall genetic diversity or marker performance among studies.

Within the present dataset, HL, MS, QLZ, TQ and WY had the highest observed haplotype counts (seven each), BMH and SCLS had the highest Hd (1.0000), and DSX had the highest nucleotide diversity (π = 0.003601). Haplotype number describes the number of sequence types observed, whereas Hd depends on their frequencies, and π reflects their average nucleotide differences per site. A population containing many closely related haplotypes therefore need not have the highest nucleotide diversity. The different rankings show that these indices describe different aspects of genetic diversity. The coexistence of widely shared haplotypes and 36 singleton haplotypes further indicates that both common sequence types and rare variants contribute to the observed diversity.

### 4.3. Chloroplast Genetic Structure

The haplotype network, sequence PCA, and exploratory clustering described chloroplast sequence variation in the present dataset from different perspectives. The K = 2 ADMIXTURE model summarized two predominant coefficient patterns, and PC1 accounted for 64.96% of the variance. The 55 haplotypes represented finer sequence variation within this broad pattern. Individuals from the same sampling population could occur in different model components, and common haplotypes were shared among multiple populations. The inferred components therefore did not correspond directly to the sampling populations. These results describe a broad pattern of sequence variation alongside overlap among sampling populations, without establishing two discrete biological populations.

Chen et al. [33] identified three chloroplast haplotype groups using network and BAPS analyses, whereas their nuclear SSR analysis identified two major components with geographic distribution patterns. The two components illustrated by the K = 2 chloroplast model in the present study differ from the three chloroplast groups previously reported. Differences in sequence regions, sampled material, and analytical methods may all influence grouping results. The number of groups alone therefore cannot establish agreement or disagreement between the two studies, nor can the descriptive K = 2 chloroplast components be directly equated with the two previously reported nuclear SSR components.

In addition, limited bootstrap support for internal NJ branches restricts interpretation of specific branching relationships. The 33 biallelic cpDNA sites used for ADMIXTURE belong to the same concatenated chloroplast sequence, so the model provides an exploratory summary rather than independent evidence for discrete ancestral populations. Shared haplotypes and network connections alone cannot quantify contemporary gene flow. The current results therefore mainly describe chloroplast sequence variation and haplotype relationships in the sampled material and provide a reference for germplasm characterization, while further evidence is needed to interpret their historical divergence.

## 5. Conclusions

Comparative plastome analysis identified localized sequence variation within a broadly conserved genomic framework, providing targets for chloroplast marker development. In 324 individuals from 37 sampling populations, the three markers yielded 55 haplotypes across 1772 complete sites (Hd = 0.8052; π = 0.002183). Marker 2 showed the highest fragment-haplotype richness and PIC. A common haplotype shared among 35 populations coexisted with 36 singleton haplotypes. Sequence PCA and the descriptive K = 2 model summarized broad patterns of chloroplast variation, while limited NJ bootstrap support constrained interpretation of specific branching relationships. These markers provide a basis for germplasm characterization and diversity assessment, although their applicability to additional material requires further evaluation.

## Figures and Tables

**Table 1:** Features of the 16 *Camellia* plastomes analyzed in this study.

Species (Sample Code)	GenBank (Accession Number)	Genome Size (bp)	LSC (bp)	SSC (bp)	IR (bp)	GC Content (%)	Total Genes	Protein-Coding Genes	tRNA	rRNA
*Camellia oleifera* (YC127)	QB224504	156,918	86,610	18,396	25,956	37.30%	132	87	37	8
*Camellia lanceoleosa* (XYYC)	QB224503	156,969	86,654	18,407	25,954	37.29%	132	87	37	8
*Camellia oleifera* (HNL2-2)	QB224502	157,003	86,656	18,297	26,025	37.29%	132	87	37	8
*Camellia* sp. XJ-2021	MN078091.1	156,981	86,647	18,284	26,025	37.28%	132	87	37	8
*Camellia sasanqua*	OL689022.1	156,545	86,257	18,402	25,943	37.32%	132	87	37	8
*Camellia vietnamensis*	OL689024.1	156,910	86,560	18,300	26,025	37.30%	132	87	37	8
*Camellia vietnamensis*	NC_060778.1	156,999	86,652	18,297	26,025	37.29%	132	87	37	8
*Camellia vietnamensis*	MN078085.1	157,089	86,627	18,132	26,165	37.28%	132	87	37	8
*Camellia vietnamensis*	MN078084.1	157,003	86,656	18,297	26,025	37.29%	132	87	37	8
*Camellia oleifera*	MN078090.1	156,939	86,644	18,407	25,944	37.29%	132	87	37	8
*Camellia oleifera*	OL689019.1	156,975	86,659	18,408	25,954	37.29%	132	87	37	8
*Camellia oleifera*	MF541730.2	156,995	86,648	18,297	26,025	37.29%	132	87	37	8
*Camellia oleifera* var. *oleifera*	MZ151356.1	156,512	86,224	18,402	25,943	37.33%	132	87	37	8
*Camellia meiocarpa*	OL689016.1	156,550	86,264	18,400	25,943	37.32%	132	87	37	8
*Camellia meiocarpa*	MN078088.1	156,512	86,224	18,402	25,943	37.33%	132	87	37	8
*Camellia gauchowensis*	OL689015.1	157,003	86,656	18,297	26,025	37.29%	132	87	37	8

**Table 2:** Target regions, expected product lengths, and polymorphism of the three chloroplast markers.

cpDNA Markers	Target Region	Expected Product (bp)	Primer Sequence (5′–3′)	Tm (°C)	Na	Ne	I	h	PIC
marker1	*trnG-UCC*	653	F: GCGGGTATAGTTTAGTGG R: TTTCATTCGGCTCCTT	55	9	1.801	0.823	0.445	0.381
marker2	*ndhF*	602	F: ATACAAGAAAAGGTAAGAAGAG R: ATCATTTTGGCAATAAGAAC	55	31	3.267	1.878	0.694	0.676
marker3	*trnL-UAA*–*trnF-GAA* and adjacent sequence	754–759	F: GTCCCTCTATCCCCAAA R: CCCATCCTTCAACTTCC	50	8	1.791	0.800	0.442	0.379
Mean	—	—		53.3	16.0	2.286	1.167	0.527	0.479

Note: Na, number of distinct fragment haplotypes; Ne, effective number of haplotypes; I, Shannon information index; h, uncorrected haplotype-frequency diversity; PIC, conventional polymorphic information content calculated from these frequencies; —, not applicable.

**Table 3:** Genetic diversity parameters of different natural populations of *C. oleifera*.

Populations	N	S	Hd	NH	π	Populations	N	S	Hd	NH	π
BMH	4	10	1.0000	4	0.002822	MS	9	11	0.9167	7	0.002790
BYS	7	8	0.6667	3	0.001935	QL	8	10	0.8929	5	0.002217
BZ	11	6	0.7273	6	0.000872	QLZ	10	10	0.9333	7	0.002370
DP	6	8	0.8667	4	0.001618	QS	12	9	0.6818	6	0.001325
DSX	7	12	0.9524	6	0.003601	QT	8	9	0.8571	5	0.002338
FJS	10	12	0.8000	5	0.002759	RX	10	2	0.6444	3	0.000665
GF	9	8	0.8056	4	0.002414	SCLS	5	10	1.0000	5	0.003273
HL	9	12	0.9167	7	0.002947	SKK	6	2	0.6000	3	0.000489
HLZ	10	2	0.6444	3	0.000477	SM	6	6	0.9333	5	0.001543
HY	6	2	0.6000	3	0.000376	SQS	8	9	0.7500	4	0.001471
JDX	7	5	0.9524	6	0.001236	TQ	17	9	0.6618	7	0.001170
JGS	11	9	0.7091	4	0.002442	WJLW	5	3	0.7000	3	0.000677
JN	8	8	0.6429	4	0.001834	WY	12	12	0.9091	7	0.002386
JXLS	7	11	0.8095	4	0.002902	WYS	8	2	0.4286	2	0.000484
JY	12	12	0.8030	6	0.002283	YLG	6	9	0.8667	4	0.002521
LC	11	5	0.6000	4	0.000852	YN	6	2	0.8000	4	0.000677
LGS	12	9	0.7273	5	0.002129	YP	11	9	0.8182	5	0.002668
LHB	6	7	0.7333	3	0.001467	ZJC	11	9	0.6000	4	0.001826
LXY	13	10	0.7179	6	0.001794						

Note: N, number of retained individuals; S, number of segregating sites; NH, number of haplotypes; Hd, haplotype diversity; π, mean pairwise nucleotide differences per site. All estimates use the same 1772 complete sites; Hd and π are displayed to four and six decimal places, respectively.

## Data Availability

The newly assembled chloroplast genome sequences have been deposited in GenBank under accession numbers QB224502 (HNL2-2), QB224503 (XYYC), and QB224504 (YC127). The raw sequencing reads generated in this study have been deposited in the NCBI Sequence Read Archive (SRA) under BioProject accession PRJNA1549989. The Supplementary File (combined_data324_trimmed2.fas) contains the trimmed concatenated marker alignment; Supplementary Table S5 provides haplotype states, frequencies, and population composition. Further inquiries can be directed to the corresponding authors.

## Supplementary Materials

The following supporting information can be downloaded at https://www.mdpi.com/article/10.3390/biology15211955/s1, Table S1: Basic information of the 37 sampled populations; Table S2: List of annotated genes in the chloroplast genomes of *Camellia* species; Table S3: Intron and exon lengths of intron-containing genes; Table S4: Numbers of SSRs by repeat motif in the 16 chloroplast genomes; Table S5: States at 38 variable sites in the 55 chloroplast haplotypes; Table S6: Codon counts and RSCU values for the three newly assembled plastomes. Figure S1: Voucher specimens of YC127, XYYC, and HNL2-2 (from left to right); File S1: combined_data324_trimmed2.fas.
